# Assessing dynamics, spatial scale, and uncertainty in task-related brain network analyses

**DOI:** 10.3389/fncom.2014.00031

**Published:** 2014-03-19

**Authors:** Emily P. Stephen, Kyle Q. Lepage, Uri T. Eden, Peter Brunner, Gerwin Schalk, Jonathan S. Brumberg, Frank H. Guenther, Mark A. Kramer

**Affiliations:** ^1^Center for Computational Neuroscience and Neural Technology, Boston UniversityBoston, MA, USA; ^2^Department of Mathematics and Statistics, Boston UniversityBoston, MA, USA; ^3^Brain-Computer Interface Research and Development Program, Wadsworth CenterAlbany, NY, USA; ^4^Department of Speech-Language-Hearing, University of KansasLawrence, KS, USA; ^5^Department of Speech, Language, and Hearing Sciences, Boston UniversityBoston, MA, USA; ^6^Department of Biomedical Engineering, Boston UniversityBoston, MA, USA

**Keywords:** functional connectivity, canonical correlation, coherence, ECoG, EEG, MEG

## Abstract

The brain is a complex network of interconnected elements, whose interactions evolve dynamically in time to cooperatively perform specific functions. A common technique to probe these interactions involves multi-sensor recordings of brain activity during a repeated task. Many techniques exist to characterize the resulting task-related activity, including establishing functional networks, which represent the statistical associations between brain areas. Although functional network inference is commonly employed to analyze neural time series data, techniques to assess the uncertainty—both in the functional network edges and the corresponding aggregate measures of network topology—are lacking. To address this, we describe a statistically principled approach for computing uncertainty in functional networks and aggregate network measures in task-related data. The approach is based on a resampling procedure that utilizes the trial structure common in experimental recordings. We show in simulations that this approach successfully identifies functional networks and associated measures of confidence emergent during a task in a variety of scenarios, including dynamically evolving networks. In addition, we describe a principled technique for establishing functional networks based on predetermined regions of interest using canonical correlation. Doing so provides additional robustness to the functional network inference. Finally, we illustrate the use of these methods on example invasive brain voltage recordings collected during an overt speech task. The general strategy described here—appropriate for static and dynamic network inference and different statistical measures of coupling—permits the evaluation of confidence in network measures in a variety of settings common to neuroscience.

## Introduction

The recent neuroscience literature has seen a dramatic increase in the number of studies that investigate functional connectivity in brain networks. Roughly speaking, functional connectivity refers to coupling (i.e., systematic associations or relationships) between neural activities in different brain regions of interest (ROIs) or recording sites (Friston, 1994; Bullmore and Sporns, 2009). Functional connectivity can be estimated from a wide range of data types with varying degrees of temporal and spatial resolution, including data with high spatial resolution but poor temporal resolution collected with positron emission tomography (PET) and functional magnetic resonance imaging (fMRI), as well as data with high temporal resolution collected using electroencephalography (EEG), electrocorticography (ECoG), and magnetoencephalography (MEG). Here, we focus on functional connectivity estimated from brain voltage recordings, i.e., EEG and ECoG (for a review of similar considerations in the context of network inference for fMRI, see Hutchison et al., 2013). One of the foremost issues associated with functional connectivity analysis is the choice of coupling measure. Coupling measures include linear and nonlinear measures of statistical association, as well as information theoretic and model based measures, and can be chosen to highlight specific types of associations such as rhythmic or causal coupling (as reviewed in Pereda et al., 2005; Greenblatt et al., 2012).

In addition to the choice of coupling measure, a number of important statistical issues arise in the inference and analysis of functional brain networks. Here we highlight three such issues. First, researchers are often interested in detecting and characterizing dynamic transitions in functional connectivity structure. Such transitions may arise suddenly as a function of a specific stimulus or behavior, or may reflect gradual ongoing changes in connectivity through time. For example, in speech production—an example we will refer to throughout this paper in order to focus our thoughts—it has been shown that functional connectivity as measured with fMRI changes during voicing (Simonyan et al., 2009), and abnormal functional connectivity has been associated with disordered speech (e.g., Chang et al., 2011). During epileptic seizure, brain functional networks assessed from ECoG data exhibit characteristic dynamical patterns that include the aggregation and fragmentation of network structure (Schindler et al., 2007; Burns et al., 2012; Kramer and Cash, 2012).

A second statistical issue in the analysis of functional connectivity in the brain concerns multiple spatial scales. At the microscopic scale, associations occur between the activities of individual neurons (e.g., Cohen and Kohn, 2011), and between the aggregate activity of small neural populations (e.g., Schnitzler and Gross, 2005). At the macroscopic scale, associations emerge between entire brain areas, or previously identified ROIs (e.g., Golfinopoulos et al., 2011). Often, the spatial scale at which brain networks are analyzed is determined by the implicit scale of the method used to record neural activity. However, there has been increasing interest in understanding relations between functional connectivity structure at multiple spatial scales. This relationship can be studied using either multiple simultaneous measures of neural activity or statistical methods that are capable of inferring associations across scales.

The final statistical issue we chose to highlight relates to estimating uncertainty in network level statistics. Typically, a selected coupling measure is estimated pairwise between all possible nodes, and the results are thresholded to produce a binary network (e.g., Stam, 2004; Micheloyannis et al., 2006; Ponten et al., 2007; Srinivas et al., 2007; Stam et al., 2007; Kramer et al., 2008; Supekar et al., 2008). Various network measures are then computed to summarize features of the resulting network topology (Kolaczyk, 2009; Rubinov and Sporns, 2010). However, the uncertainty pertaining to these network level statistics is typically not computed. This uncertainty will be related to the uncertainty associated with each of the pairwise connections, but propagating this edge uncertainty to network level uncertainty is nontrivial and remains an active area of research.

In this paper, we present a framework for inferring functional connectivity that addresses each of these issues. After introducing a statistical methodology appropriate for analyzing connectivity in a given time window with respect to a baseline condition (Methods sections Construction of Functional Networks and Assessing Uncertainty), we demonstrate the use of this network methodology in the context of assessing dynamics, spatial scale, and uncertainty in networks. First, temporally dynamic changes in network structure are tracked (Results section Dynamics). Second, using a priori spatial information, functional connectivity is generalized from between-node connectivity to between-region connectivity. This procedure reduces the number of inferred connections relative to the number of measurements, and trades spatial resolution for a more reliable estimate of functional connectivity. We apply both of these approaches to ECoG data collected during an overt speech task for one subject (Results section ECoG Data), demonstrating their feasibility and highlighting some desirable features. Finally, we present several examples to illustrate advantages of the canonical correlation metric (Results section Advantages of the Canonical Correlation Measure). Throughout these discussions we quantify the uncertainty inherent in estimates of functional connectivity (Methods sections Dynamics, ECoG Data, and Advantages of the Canonical Correlation Measure). Together these approaches advance the burgeoning field of functional connectivity in important ways and provide a general tool for the construction of meaningful functional networks in task-related data with associated measures of confidence. The paper concludes with a discussion of limitations and of future research directions.

## Methods

Network analyses take a wide variety of forms; even for functional networks constructed using cross correlation (i.e., correlation-based networks) applied to brain voltage data, there are a number of design decisions that affect the interpretation of the resulting networks. Here we focus on how correlation-based networks differ during a task compared to a baseline period, and we note that the same framework developed here also applies to other choices of coupling measure, including coherence-based networks (see Discussion). We propose a multi-step analysis strategy that builds on previous work on edge-count uncertainty in binary networks (Kramer et al., 2009), utilizing the trial structure of the data to increase power, highlight differences between task and baseline, and assess uncertainty. In brief, the proposed method determines network edges (between individual sensors or groups of sensors collected across ROIs) using a statistical hypothesis test, corrects for multiple comparisons, and computes confidence measures at the single edge and aggregate network measure levels. As described in detail below, nonparametric bootstrap estimation (Efron and Tibshirani, 1993) is used both for the statistical hypothesis test to detect individual edges, and for the determination of confidence intervals over edges and aggregate network measures. Because bootstrapping is nonparametric, it requires minimal assumptions about the data.

### Motivation: speech task

Here we will consider networks constructed during speech production as compared to periods of silence. While this example will be used throughout the paper, any repeated task with a baseline period could be substituted. The production of speech involves a large network of brain regions that spans several lobes of the cerebral cortex along with numerous subcortical structures (e.g., Guenther et al., 2006). Fluent speech requires very rapid movements of the tongue and other articulators. For example, a typical speaker can easily produce 10 phonemes in 1 s; this involves the precise sequencing of individual articulatory gestures that each last approximately 100 ms. The most commonly used neuroimaging techniques for studying speech, PET and fMRI, have a temporal resolution on the order of 1 s, precluding them from measuring these rapid dynamical processes. Furthermore, speech articulation creates massive muscle-related artifacts in EEG and MEG, limiting the utility of these technologies for studying speech production. To overcome these problems, a number of neuroscientists have begun studying speech using ECoG recordings collected prior to epilepsy surgery (e.g., Korzeniewska et al., 2011; Pei et al., 2011; Leuthardt et al., 2012; Bouchard et al., 2013); ECoG recordings have high temporal resolution (typically on the order of 1 ms) combined with reasonably high spatial resolution (on the order of 1–10 mm on the cortical surface). Thus, ECoG offers the possibility of studying dynamic changes of functional connectivity within the speech network, potentially providing a powerful tool for deciphering the neural dynamics underlying speech and other actions or cognitive tasks.

In general, quite complex topologies are derived using modern neuroimaging techniques. For example, a network consisting of 100 electrodes (typical in both noninvasive and invasive brain voltage recordings) may contain up to 4950 edges. Many measures exist for assessing the organization of these edges (Kolaczyk, 2009; Rubinov and Sporns, 2010). However, principled methods to determine confidence in these network measures are not well established. A notion of confidence is particularly important when assessing how networks change in time. For example, during a reading task, we may ask how the functional network changes during the different stages of the task, starting from visual and linguistic processing of the stimulus, proceeding to motor planning and execution, and finally involving the processing of auditory and somatosensory feedback during speech. In the simplest scenario that two functional networks are established—one preceding the onset of speech, and the other following—we might then ask how the density, a network measure defined as the ratio of the number of detected edges to the number of possible edges in the network, differs before and after speech onset. To answer this question in a meaningful way requires a measure of uncertainty in the density. In what follows, we propose a resampling procedure to estimate the sampling variability of individual edges as well as to establish confidence intervals for density and other aggregate network measures.

### Simulated data

Simulated data were generated to mimic ECoG recordings in which a task is performed multiple times, and the resulting brain activity observed. For example, a speech task may involve showing the subject a specific word, which the subject would read multiple times during the experimental session. In general, we expect the pattern of correlations between electrodes to vary as a function of time with respect to task onset. If the task onset is defined as the start of the behavioral response (e.g., overt speech), there may be task-related activity and connectivity that precede the task onset, such as processing of the visual stimulus and motor preparation. Hence we consider the trials to start before task onset and last until some time after task onset (specifically, 500 ms before until 500 ms after task onset).

**Figure 1:**
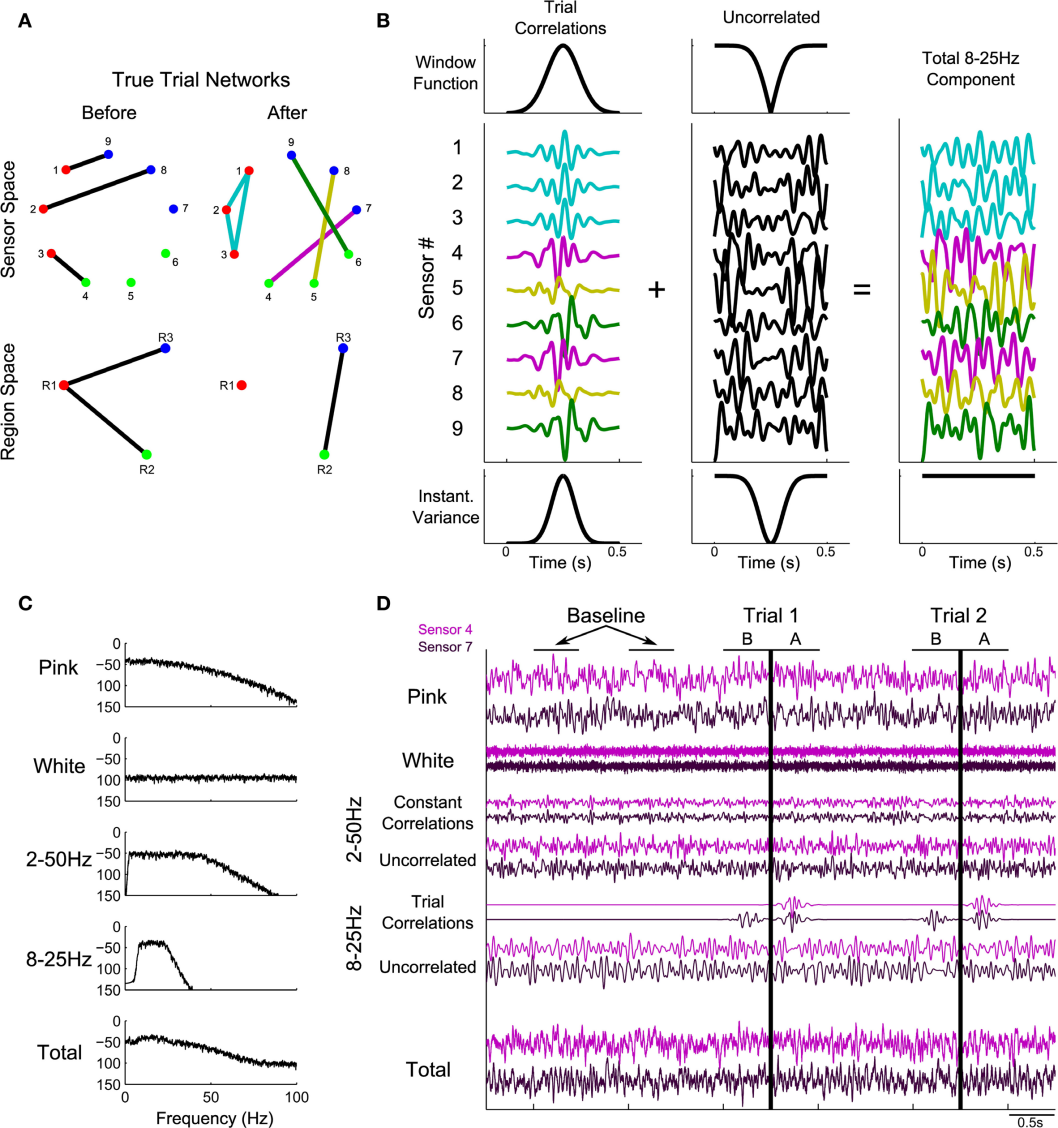
**Illustration of construction of the simulated data**. Predetermined networks **(A)** were chosen for periods before and after task onset. Sensor-space networks (**A**, top row) correspond to region-space networks (**A**, bottom row). Node colors correspond to region assignments. **(B)** shows how the correlation structure defined in **(A)** was introduced into the simulated data, using the after network to illustrate. The correlation structure in the 500 ms of each trial following task onset consisted of four instantiations of 8–25 Hz noise (**B**, left), added to the sensors according to the defined network (colors in **B** correspond to the edge colors in the after network in **A**). The trial correlations were introduced into a background of uncorrelated 8–25 Hz noise by windowing (**B**, top row) in such a way that the variance remained constant in the total signal (**B**, bottom row). In addition to the 8–25 Hz component, three other signals were added: pink noise, white noise, and a 2–50 Hz signal containing a correlated and uncorrelated part (**C**: power spectra; **D**: time domain). **(D)** shows sample data for 2.5 s of baseline and two trials. Vertical black lines correspond to task onset: the before network is in effect for 500 ms before this time and the after network is in effect for 500 ms after this time. For more details regarding the construction of simulated data, see Methods section Simulated Data and Appendix section Construction of Simulated Data.

In the simulations performed here, we consider dynamic activity recorded from nine sensors. The synthetic data at each sensor consist of four dynamic components with a known pattern of correlations. The first component, pink noise, captures one feature of brain voltage activity, the “1/f” reduction in power as a function of frequency common in brain voltage activity (Miller et al., 2009; He et al., 2010). The second component is white noise, meant to represent sensor noise. On top of these two uncorrelated components, two types of correlated signal are added: 2–50 Hz correlations that exist throughout the recording, and 8–25 Hz correlations that only come into effect during the trials. The first is meant to represent any persistent correlated structure existing in the signals that does not change during the trials, such as correlations related to the recording apparatus. The trial correlations are meant to represent task-related changes in the correlation structure. For example, during a speech task there may be increased correlations between areas involved in speech, including high-level motor areas, primary motor cortex, and auditory and somatosensory cortices (Guenther et al., 2006). While in a true task this structure may change many times during the different stages of task execution, in the simulated data we introduce only two known network structures: one that gradually increases, peaks, and fades away in the 500 ms before task onset on every trial, and one that gradually increases, peaks, and fades away in the 500 ms after task onset on every trial. Details of these networks and how the simulated data were generated are described in Appendix (section Construction of Simulated Data) and summarized in Figure 1.

### ECoG data

To illustrate the use of the methods on experimental data, we analyze ECoG time series recorded during overt speech.

#### Experimental protocol and ECoG recording

The neuronal recordings were collected from a single individual undergoing treatment for intractable epilepsy involving implantation of subdural electrocorticographic grids, used for localization of seizure foci for later resection of epileptic tissue. The electrodes consisted of two, 1-cm spaced grids positioned over the frontal and parietal lobes, and the temporal lobe, respectively, and a strip of electrodes over the occipital lobe. Signals were recorded using g.tec g.USBamp amplifiers (sampling rate 1200 Hz). Data acquisition and stimulus presentation were handled using BCI2000 software (Schalk et al., 2004).

During the task the subject read the Gettysburg Address (272 words) aloud from a video monitor, as the text scrolled from right to left. The full session lasted 295 s. There were no seizures during the experimental session, and electrodes near the putative seizure focus were excluded from the analysis. The subject gave informed consent to participate, and this research has been approved by the local institutional review boards.

#### Preprocessing of ECoG data

The analyses were based on recordings from 90 electrodes. The slow progression of the teleprompter forced the subject to pause periodically, and these pauses were used to define a trial structure. Specifically, trials were defined as any time during the speech recording where 500 ms of silence was followed by at least 500 ms of speech, determined using an audio recording of the session. Hence, each trial lasted 1 s, and contained activity related to reading the word(s), preparing the motor command, and voicing the word or phrase aloud. Note that the specific words spoken during the trials varied. There were a total of 98 trials so defined. Furthermore, data from silences separated by more than 500 ms from the beginning or end of any utterance were extracted to be used in defining the baseline distribution, described below (section Assessing the Significance of the Test Statistic).

#### ROI definitions

ROIs were defined anatomically for the subject based on manual inspection of structural MRI by an experienced anatomist. The 25 regions corresponded to anterior/middle dorsal premotor cortex, posterior middle frontal gyrus, inferior frontal gyrus, pars opercularis, posterior dorsal premotor cortex, middle premotor cortex, ventral premotor cortex, dorsal primary motor cortex, middle primary motor cortex, ventral primary motor cortex, dorsal somatosensory cortex, middle somatosensory cortex, ventral somatosensory cortex, superior parietal lobule, anterior supramarginal gyrus, posterior supramarginal gyrus, fronto-orbital cortex, temporal pole, anterior superior temporal gyrus, posterior superior temporal gyrus, anterior middle temporal gyrus, posterior middle temporal gyrus, anterior inferior temporal gyrus, posterior inferior temporal gyrus, ventral temporal cortex, and occipital cortex (Golfinopoulos et al., 2011). Electrode ROI assignments are indicated by node color in Figure 9.

### Construction of functional networks

**Figure 2:**
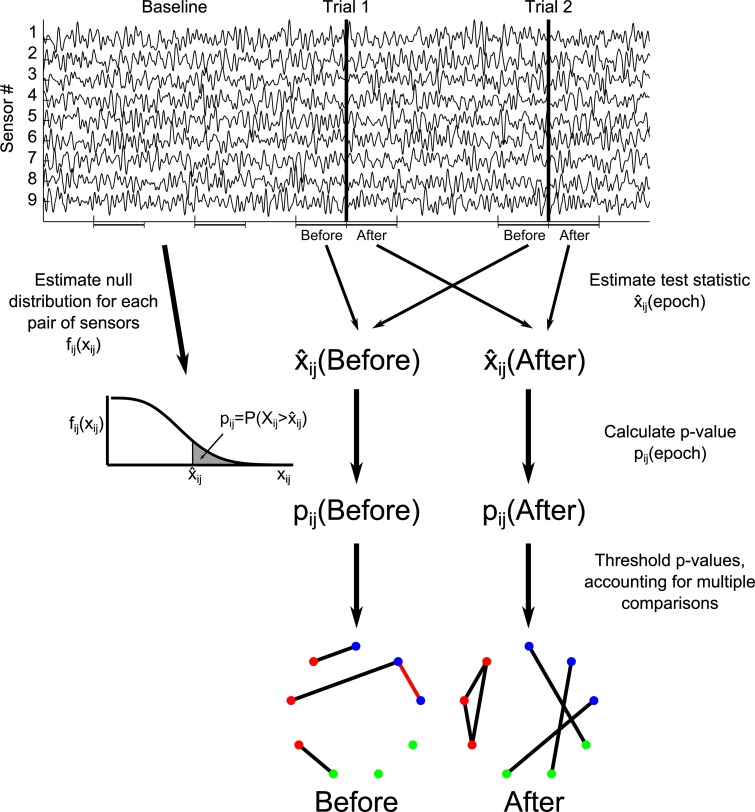
**Illustration of construction of functional networks from the time series data**. For each trial epoch of interest (here labeled “Before” and “After”), the test statistic $\hat{x}$_*ij*_ for each potential network edge (between node *i* and node *j*) is calculated over all trials. The test statistic is compared to a null distribution estimated from baseline intervals, resulting in *p*-values for each potential edge, *p*_*ij*_. A threshold is chosen for the *p*-values using a multiple comparisons correction, resulting in binary networks. Red edges in the networks indicate false positive edges, identified by comparison to the true networks. For more detail, see Methods section Construction of Functional Networks.

The specific method for inferring functional network structure from brain voltage recordings used here contains a set of techniques for analyzing the dynamics, spatial scale, and uncertainty in network connectivity. While we discuss the behavior of this technique as a whole, the steps are modular and generally applicable: for example, the coupling statistic could be changed from correlation to coherence or phase locking value while keeping the other aspects of the analysis, like uncertainty estimation, intact. Alternatively, a different technique for correcting for multiple comparisons could be used to identify binary edge assignments. Below, we describe our choices for each module of the functional network analysis: preprocessing (including defining trial epochs), choice of test statistic, *p*-value calculation through comparison to a null distribution, multiple comparisons correction, and uncertainty assessment. This procedure is also outlined in Figure 2. One additional module, the selection of network nodes, is implicit in this analysis. We use two different node definitions corresponding to the coupling statistics, defining each node as an individual sensor (for correlation) or all of the sensors in an ROI (for canonical correlation).

#### Preprocessing of the data

To infer functional networks from the synthetic time series, the data were first preprocessed by band-pass filtering between 0.1 and 30 Hz using a zero-phase 3rd order Butterworth filter (Matlab filtfilt function). This preprocessing step was performed to mimic the typical procedure of bandpass filtering observed brain voltage recordings to eliminate artifacts and focus on a particular frequency range. The data were then downsampled to 200 Hz. For the ECoG data, a common average reference was applied by subtracting the mean over all channels from each channel at each time step. This step was intended to reduce referencing effects from the experimental signals.

In order to analyze time-varying functional connectivity, the trial data were then divided into epochs of interest spanning the 1s-long trials. Two kinds of epoch were defined. The first were 500 ms non-overlapping epochs consisting of the first and second halves of the trials (500 ms each, with no overlap), corresponding to “before” and “after” task onset, defined as time zero. The second kind of epoch used a 200 ms sliding window (195 ms overlap) over trials, allowing for the construction of “dynamic” networks on overlapping time points starting from 400 ms before time zero until 400 ms after time zero (the 200 ms epochs were labeled according to their time midpoints).

The epochs of interest were collected from all trials resulting in a 4-dimensional matrix with dimensions (*E* × *T* × *N* × *L*), where *E* is the number of epochs, *T* is the number of time points per epoch, *N* is the number of sensors, and *L* is the number of trials. For example, using before/after epochs in the simulations here, there were two epochs, each consisting of 100 time points (corresponding to 500 ms sampled at 200 Hz), nine sensors, and 100 trials. Using dynamic epochs, there were 162 epochs, 40 time points per epoch (200 ms sampled at 200 Hz), nine sensors, and 100 trials. The data from each sensor, trial, and epoch were normalized by subtracting out the mean and dividing by the standard deviation.

For each epoch type, a group of non-overlapping “baseline” intervals were also collected from the baseline period for characterization of the null distribution of the test statistic between each pair of sensors. We note that the baseline intervals contain no trial data and hence should not contain task-related correlated activity, yet may possess other correlated activity due to the persistent correlation signal common to all sensors. The intervals of baseline data were chosen to have the same duration as the trial epochs, and were stored in a 3-dimensional matrix with dimensions (*T* × *N* × *K*), where *K* is the number of baseline intervals. For example, for before/after epochs in the simulated data, 400 non-overlapping intervals of the (preprocessed) baseline data, 100 time points long, were collected from the baseline period. Note that *K* does not need to be the same as *L*: having more baseline intervals than trials will improve the estimation of the null distribution (described in section Assessing the Significance of the Test Statistic). The baseline intervals are normalized in the same way as the trial intervals, by subtracting the mean and dividing by the standard deviation.

#### Computing the test statistic

Two measures of coupling (test statistics) were employed to establish the functional networks: correlation and canonical correlation. For a given epoch, the test statistics were calculated for each pair of nodes using all trials. Hence calculating the test statistics resulted in a 3-dimensional matrix with dimensions (*E* × *N* × *N*) for sensor-space networks and (*E* × *R* × *R*) for region-space networks, where *R* is the number of regions.

We focus on correlations that become more extreme (more positive or more negative) during the task, so the absolute value of the raw correlation values was used as the sensor-level test statistic. The absolute value of the correlation was calculated as:

$$AbsCorr(x,\;y)=\left|\frac{\sum_{l=1}^{L}\sum_{t=1}^{T}x_{l}(t)y_{l}(t)}{\sqrt{\left(\sum_{l=1}^{L}\sum_{t=1}^{T}x_{l}{\left(t\right)}^{2}\right)\left(\sum_{l=1}^{L}\sum_{t=1}^{T}y_{l}{\left(t\right)}^{2}\right)}}\right|$$

where *x*_*l*_(*t*) and *y*_*l*_(*t*) are the voltage at time *t* of the given epoch of trial *l* for sensors *x* and *y*, respectively.

Canonical correlation was chosen as a region-level coupling measure because of its relationship to the sensor-level correlations. A common measure of network topology (used to describe networks that have already been constructed) involves the identification of groups of nodes (e.g., communities). However, when analyzing multivariate data recorded from the brain, there often exists an a priori natural grouping of nodes belonging to a region of interest (e.g., a brain region associated with a particular function). By using a classical statistical multivariate technique—canonical correlation—connectivity between groups of channels can be studied in a principled way. Canonical correlation finds the linear combinations of the group members such that the linear combinations are maximally correlated. Canonical correlation is reported in classical texts on multivariate statistics (e.g., Mardia et al., 1979; Anderson, 2003). When a priori knowledge of signal presence on group member channels is not known, canonical correlation possesses increased statistical power and is a powerful method of dimensionality reduction in the context of network analysis. This is discussed further in Results section Advantages of the Canonical Correlation Measure.

Formally, the canonical correlation between two groups of signals $\vec{x}$ = (*x*_1_, …, *x*_*n*_)^*T*^ and $\vec{y}$ = (*y*_1_, …, *y*_*m*_)^*T*^ seeks to find linear combinations of $\vec{x}$ and $\vec{y}$ that are maximally correlated:

$$CanonCorr(\vec{x},\;\vec{y})=\underset{\vec{a},\vec{b}}{\max}\;Corr\left({\vec{a}}^{T}\vec{x},\;{\vec{b}}^{T}\vec{y}\right)$$

For convenience, we will switch to matrix notation: *X* and *Y* are (*n* × *LT*) and (*m* × *LT*) matrices, respectively, containing a row of appended trial voltages for each sensor in the region. The optimization problem for canonical correlation is solved using the singular value decompositions (Strang, 2003) of *X* and *Y*, *X* = *U*_*X*_ Σ_*X*_ *V*^†^_*x*_ and *Y* = *U*_*Y*_ Σ_*Y*_ *V*^†^_*Y*_. The canonical correlation is the first singular value of the matrix *Q*_*XY*_ = *U*_*X*_ *V*^†^_*X*_ *V*_*Y*_ *U*^†^_*Y*_.

#### Assessing the significance of the test statistic

After computing the test statistic between each pair of nodes, a *p*-value is computed using the null hypothesis that the test statistic for that edge comes from the same distribution as the baseline period (which lacks task-related activity). The alternative hypothesis is that the test statistic is too large to be from the baseline distribution. For example, in our analysis of speech data we use a null distribution estimated from epochs of time without speech (i.e., silence). Note that for simplicity we have chosen a one-sided hypothesis test, focusing only on correlations or canonical correlations that are larger than baseline. All of the methods described here could be developed for a two-sided hypothesis test, allowing for both higher and lower correlations and canonical correlations than baseline.

In order to estimate the null distribution, a bootstrap procedure is employed involving resampling of the baseline data. In particular, for each bootstrap sample, *L* baseline intervals (described in section Preprocessing of the Data) are sampled with replacement from the *K* total baseline intervals. The test statistic for each potential edge is calculated from these baseline intervals just as it was for the set of trial intervals. Here, 1000 bootstrap samples were processed in this way, thus building an empirical distribution for the test statistic from the baseline period for each potential edge.

In order to determine a *p*-value associated with each edge, the test statistic is computed during the epoch of interest and compared to the null distribution. Specifically, the *p*-value associated with the edge between node *i* and node *j* is calculated as the number of samples in the bootstrapped empirical distribution for nodes *i* and *j* that fall above the observed test statistic between nodes *i* and *j*, divided by the total number of bootstrap samples (here we use 1000 samples for simulated data, and more for the real data: for discussion, see Appendix section Sparsity of Networks and Null Distribution Resolution Effects). When the test statistic is larger than any of the samples in the empirical distribution, this method assigns a *p*-value of zero, which is unrealistic. To account for this, we set all zero *p*-values to the smallest possible *p*-value for the number of bootstrap samples (e.g., 1/1000 for the simulated data).

#### Correcting for multiple comparisons

After a *p*-value has been computed for each potential edge, a correction for multiple comparisons is performed using the Benjamini–Hochberg procedure to control for False Discovery Rate (hereafter referred to as the FDR procedure; (Benjamini and Hochberg, 1995)). This procedure sets a theoretical limit, *q* (here, 0.05), on the expected number of falsely detected edges as a proportion of the total number of detected edges. The procedure first sorts the *p*-values in increasing order and then chooses the highest integer *k* such that:

$$p_{\left(k\right)}\leq \frac{qk}{N_{MC}}$$

where *N*_*MC*_ is the total number of comparisons being performed, and *p*_(*k*)_ is the *k*^*th*^ smallest *p*-value. The FDR procedure thus selects a set of edges to consider significant such that only 5% of the detected edges are expected to be false positives. In contrast, a typical *p*-value threshold of α = 0.05 implies that 5% of all possible edges will be expected to be false. The FDR procedure is thus expected to have many fewer false detections than would occur with a 5% *p*-value threshold. Note that when the null distribution is estimated from data, as it is here, more than the expected number of false positives may occur in the network due to sampling bias (Dudoit and Laan, 2008).

The resampling approach used here to estimate the baseline null distributions, combined with the FDR procedure for multiple comparisons correction, imposes a lower bound on the number of edges that can be detected in the networks. This lower bound scales with the square of the number of nodes in the networks, so it can become large for a network with many nodes. This phenomenon and some approaches to dealing with it are discussed more in the Appendix (section Sparsity of Networks and Null Distribution Resolution Effects).

After the FDR procedure has been applied, the resulting assessment of test statistics as significant or not significant is used to define a binary network for the epoch of interest. This procedure is repeated for all epochs of interest (e.g., 500 ms before and after time zero), creating a dynamic series of networks. Note that the null distributions do not need to be estimated separately for each epoch. The null hypothesis is defined in terms of baseline distributions for each potential edge that are independent of trial epoch, so the same estimated distributions can be used for all epochs. The procedure for constructing a functional network is also illustrated in Figure 2.

### Assessing uncertainty

We focus on assessing the uncertainty of two network features: (1) edge existence, and (2) network density—the total number of edges in a network divided by the number of possible edges. To do so, we employ a classic nonparametric bootstrap procedure, making use of the trial-structure of the data (Efron and Tibshirani, 1993). Specifically, the time series data from the trials are resampled with replacement, the test statistic at each potential edge is calculated over the resampled trials, *p*-values are computed by comparison of the test statistic to the same baseline distribution used above, the *p*-values are thresholded using FDR to create a binary network, and an aggregate network measure (the density) is computed for the network. This process of resampling generates a population of surrogate networks (here we use 100 bootstrap samples) that can be used to approximate the true network population. For example, the probability of each edge can be estimated as the proportion of the surrogate networks that contain that edge. Statistically, this approach treats each potential edge as a Bernoulli random variable with parameter *p*, the probability of “success,” or the probability of observing an edge. The variance is therefore equal to *p*(1 − *p*). Hence, estimating the probability of an edge allows us to assess the variability of the network edges themselves.

For an aggregate network statistic such as the density, the distribution of the statistic calculated on the resampled networks is an estimate of the true sampling distribution of the statistic. Here, we estimate the standard error of the density as the standard deviation of the resampled densities, *ŝ*. Then, a 95% confidence interval around the observed network density *d*_*obs*_ is constructed using a Gaussian approximation as [*d*_*obs*_ − 1.96*ŝ*, *d*_*obs*_ + 1.96*ŝ*].

The estimates of the sampling distributions of networks and network statistics constructed this way are biased because each surrogate network is constructed using fewer unique trials than the original network (constructed using all trials). This results in decreased degrees of freedom in the surrogate networks, which has the effect of increasing the occurrence of false positives. This is similar to training-set-size bias that occurs in prediction error estimation (Hastie et al., 2009). Increasing the number of false positives will tend to increase the probability of individual edges and the overall density of the surrogate networks relative to the original network. Hence, (1) the estimated probabilities of edges reported below will have a small upward bias, and (2) the full bootstrap distribution of densities estimated as described above will be biased upward. Note that we chose to construct confidence intervals of the density using only the standard error of the bootstrap distribution, in part to mitigate this issue. Alternative approaches exist, including the construction of confidence intervals using quantiles of the bootstrap distribution, possibly correcting for the bias using a technique such as the “Bias-Corrected and Accelerated” method (BCa) (Efron and Tibshirani, 1993).

See Appendix (section Network Inference Algorithm Structure Including Uncertainty Estimation) for the full algorithm.

## Results

In this section we apply the network inference procedure described above to construct functional networks from synthetic data. We consider different simulation scenarios to illustrate the utility of the method in assessing the dynamic evolution of networks, in the principled spatial aggregation of network nodes using canonical correlation, and in the determination of uncertainty in network edges and measures.

### Dynamics

#### Inference of functional networks before and after task onset

**Figure 3:**
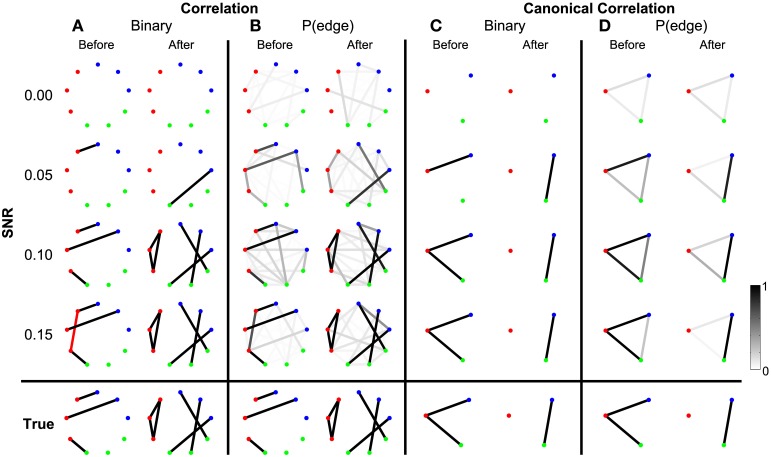
**Detected networks reflect the true correlation structure for sufficiently high SNR**. Correlation and canonical correlation networks are shown before and after task onset, for simulations with varying SNR. **(A)** Binary correlation networks for 500 ms before task onset (left) and the 500 ms after task onset (right). True positive edges are colored black, false positive edges are colored red. **(B)** Bootstrapped probability of each edge for the correlation before (left) and after (right) networks. Probabilities are indicated in grayscale from 0 (white) to 1 (black); scale bar at bottom right of the figure. **(C)** The same as **(A)**, but for canonical correlations between regions. **(D)** The same as **(B)**, but for canonical correlations between regions. Plots **(A–D)** are shown in the rows for four different levels of SNR: 0.00, 0.05, 0.10, and 0.15. True networks are shown in the bottom row. In all networks, nodes are color-coded according to region.

To begin we consider the scenario in which the only coupled activity between electrodes appears during trials. Outside of these time intervals, the simulated activity for each electrode consists only of uncorrelated noise; in these simulations, no correlated baseline activity exists between the electrodes (i.e., the constant correlations were set to zero, see Methods section Simulated Data and Appendix section Constant Correlations). We perform these simulations for four different levels of signal-to-noise ratio (SNR). Functional networks were constructed using both the correlation and canonical correlation for these simulated data in two intervals: one interval immediately before the onset of the task (the first half of the trial), and another immediately after the onset of the task (the second half of the trial); both intervals are of duration 0.5 s. As expected, the network inference procedure identifies the appearance of the correct functional network before and after task onset when the SNR is sufficiently large. This occurs for networks computed using both correlation (Figure 3A) and canonical correlation (Figure 3C). Note that for the correlation networks, edges appear between the (nine) individual nodes, while for the canonical correlation networks, edges appear between the (three) ROIs.

In addition to the binary network inference, the proposed method permits a characterization of the confidence in each edge. For both the correlation and canonical correlation networks, we associate with each edge a probability of appearance (determined through resampling, see Methods section Assessing Uncertainty). As expected, edges that appear in the binary networks possess a high probability of appearance in the correlation (Figure 3B) and canonical correlation (Figure 3D) networks. These results illustrate that the network inference procedure—and associated measures of edge uncertainty—accurately identify the underlying network when no coupled baseline activity exists between the nodes, even when the SNR is relatively small (0.1).

**Figure 4:**
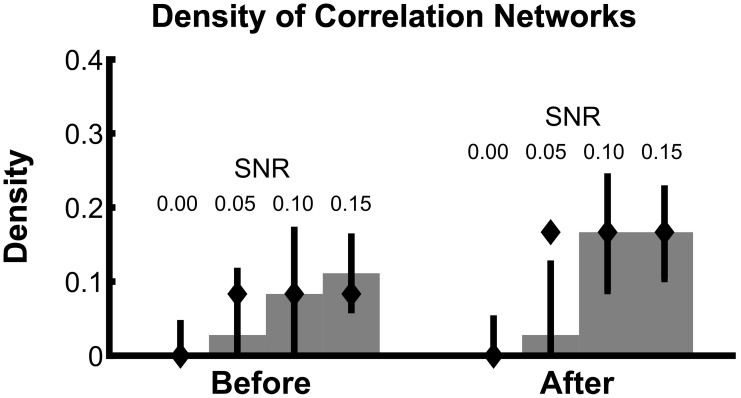
**Density estimates improve with increasing SNR**. Density of bootstrapped correlation networks in the before and after trial epochs are shown, for simulations with four different SNRs: 0.00, 0.05, 0.10, and 0.15. For details of the bootstrapping procedure, see Methods section Assessing Uncertainty. Bar height indicates the observed density, and whiskers indicate 95% bootstrapped confidence intervals. The true values of the density in the before and after networks are indicated with diamonds.

To summarize how the network topology changes from the interval before task onset to the interval after task onset, we compute a fundamental network measure: the density (see Methods). For the true network, the density is 3/36 = 0.083 before task onset, and 6/36 = 0.167 after task onset. At each level of SNR, the density for the correlation networks is easily computed from the resulting binary networks in Figure 3A (SNR = 0.00, before = 0, after = 0; SNR = 0.05, before = 0.028, after = 0.028; SNR = 0.10, before = 0.083, after = 0.167; SNR = 0.15, before = 0.111, after = 0.167). In addition to this single density value, we also determine the confidence interval for each density value (Figure 4). To compute these confidence intervals, we use the resampling procedure described in Methods section Assessing Uncertainty. We find that, as the SNR increases, the density before and after task onset also increases. In addition, at larger SNRs (namely, SNR = 0.10, and SNR = 0.15), the confidence intervals for the densities of the inferred correlation networks include the known true network densities (Figure 4). The confidence intervals at an SNR of 0.05 do not enclose the true network density value after task onset, highlighting the fact that the estimated confidence intervals may themselves be biased, here toward zero, and are variable due to the limited sample of trials used to estimate them. In summary, this approach provides a technique to calculate not only the aggregate network measure (density), but also an associated level of confidence. The results for the canonical correlation networks are similar (in this case, the true network density is 2/3 = 0.66 before task onset and 1/3 = 0.33 after task onset) and are not included here.

**Figure 5:**
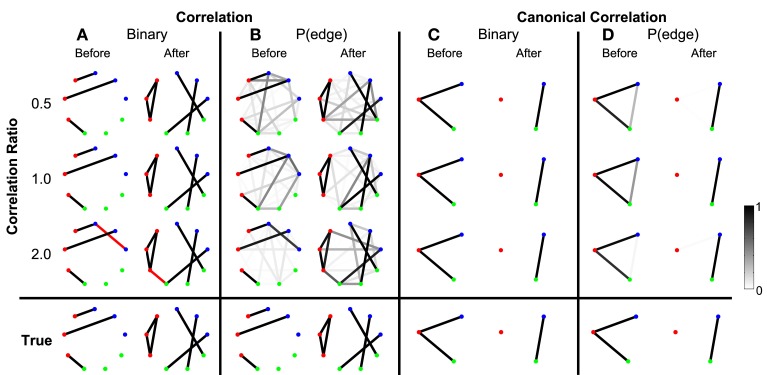
**True networks can be detected in the presence of background correlations**. Correlation and canonical correlation networks are shown before and after task onset, for simulations with varying correlation ratio. **(A–D)** as in Figure 3, shown in the rows for three different levels of the ratio of trial correlation variance to constant correlation variance: 0.5, 1.0, and 2.0. All simulations have a fixed SNR of 0.11. False positives are indicated in **(A,C)** by red edges. True positives are displayed in black.

Next we consider how the network inference procedure performs when additional coupling not related to the task exists in the data. This “constant” coupling corresponds to persistent correlations between the nodes that occur throughout the recording, i.e., both in the baseline periods and during the trials (described in Methods section Simulated Data and Appendix section Constant Correlations). The simulations varied by “correlation ratio,” which we define to be the ratio of the variance of the correlated trial component to the variance of the constant correlated component. Hence a small value of the correlation ratio represents weaker trial correlations relative to background (nuisance) correlations (see Appendix section Construction of Simulated Data for more detail). We apply the network inference procedure to the data, and find good performance (Figure 5); the inferred binary networks agree with the true networks. Interestingly, spurious edges do not appear in the binary correlation networks with trial correlations equal to or lower than constant correlations (ratios 1.0 and 0.5, respectively), but do appear in the simulation in which the trial correlations were twice as strong as the constant correlations (red lines in Figure 5A, for correlation ratio of 2.0). Computing the probability of each edge shows that the spurious edges possess lower probability of appearance, while the true network edges are highly probable (Figure 5B). In this way, determination of the edge probability provides additional information to help identify spurious network edges. We note that, for the canonical correlation, these spurious edges are not present. Instead, the appropriate edges between the ROIs are correctly identified (Figure 5C), with high probability (Figure 5D). In this way the canonical correlation measure provides additional robustness to the appearance of spurious edges between individual node pairs.

**Figure 6:**
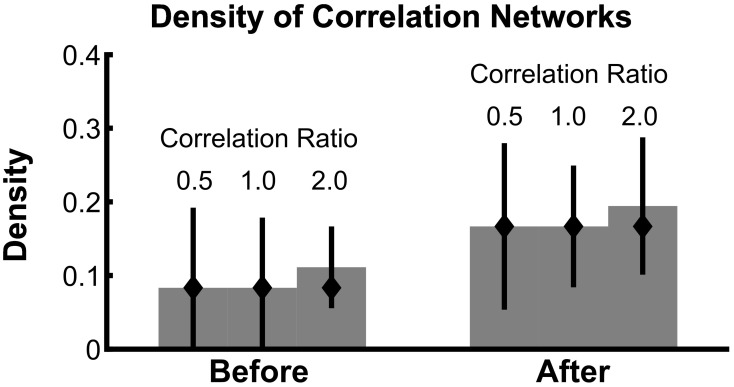
**Confidence intervals on network density include the true density, even in the presence of baseline correlations**. The density of bootstrapped correlation networks is plotted before and after task onset, for simulations with three different levels of the correlation ratio: 0.5, 1.0, and 2.0. Bars, confidence intervals, and true density as in Figure 4.

Analysis of the correlation network density in the presence of constant correlations (Figure 6) reveals that the 95% confidence intervals of the density contain the true density in all cases, even when the observed network contains false positives. These results show that even for weak values of trial-specific correlation (compared to constant correlation), the estimate of network density is accurate.

We note that these simulation results correspond to a single instantiation of the synthetic time series data consisting of multiple trials. We focus only on a single instantiation to mimic the typical experimental paradigm, in which an individual subject performs the experiment once. Repeating the analysis for different instantiations (i.e., with different values of noise) we find similar qualitative results: namely, that the network inference procedure correctly identifies the true underlying network and changes in density (results not shown). What differs across these instantiations is both the number and location of false positive and false negative edges.

#### Inference of dynamic functional networks

**Figure 7:**
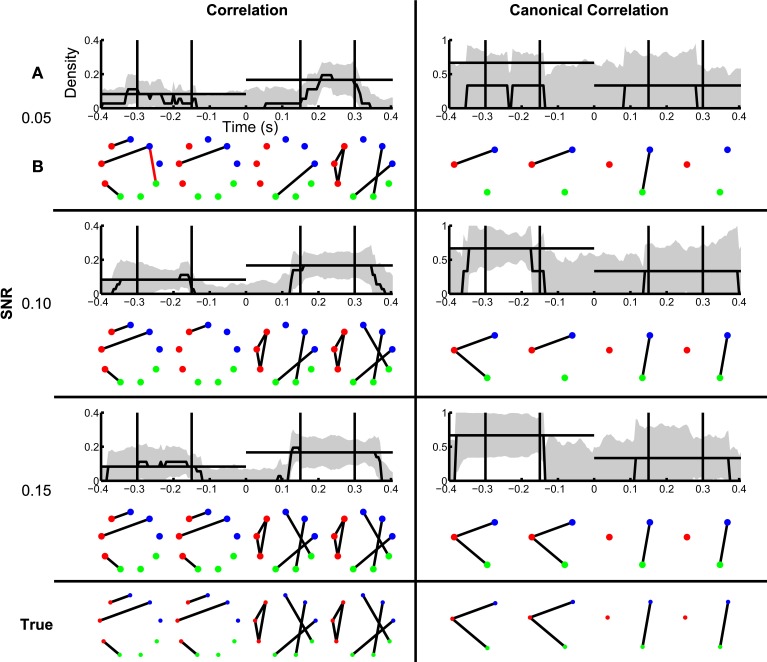
**Dynamic changes in network structure are better resolved at higher SNRs**. Correlation and canonical correlation networks are shown as a function of time with respect to task onset, for simulations with varying SNR. **(A)** Density of observed networks as a function of time (black), with 95% bootstrapped confidence intervals (shaded gray). The timing of four example binary networks **(B)** is indicated by black vertical lines, and the true densities are indicated by horizontal black lines. In **(B)**, network edges that exist in the true network are shown in black and false positive edges are shown in red. In all networks, nodes are color-coded according to region. **(A,B)** are shown for correlation (left) and canonical correlation (right) networks, for three different levels of SNR: 0.05 (top), 0.10 (middle), and 0.15 (bottom). True networks are shown in the bottom row.

In section Inference of Functional Networks Before and After Task Onset, we illustrated how the inferred functional networks changed from an interval immediately preceding task onset, to an interval immediately after task onset. We now further explore the applicability of the network inference procedure to dynamic (i.e., time-indexed) networks. To do so, we consider the same task-related data, but infer functional networks from overlapping windows of duration 0.2 s (0.195 s overlap) that begin 0.4 s before task onset, and end 0.4 s after task onset (networks are labeled by their midpoint, e.g., the network at time −0.4 s represents data from −0.5 s until −0.3 s). Task onset is defined to be time 0 s. Within each window, we apply the network inference procedure described above, and compute a binary network and the density with an associated confidence interval. We show the resulting dynamic correlation and canonical correlation networks for increasing SNR in Figure 7. For comparison, we show both the dynamic density estimate with confidence intervals (Figure 7A) and example inferred functional networks (Figure 7B) for each value of SNR. In both cases, as the SNR increases, the inferred functional networks more accurately capture the true networks. We note that, even for low SNR (=0.05), a significantly non-zero increase in density appears following task onset for the correlation networks. However, before task onset the confidence intervals rarely achieve significantly nonzero density. We note that the bootstrap density distributions tend to exceed the true density and possess long tails due to the occurrence of false positive edges (not shown). This leads to a large standard error that is relatively insensitive to SNR, and confidence intervals that include zero in this procedure. The same issue arises in the canonical correlation networks after task onset for high SNR. An alternative approach based on quantiles of the bootstrap distributions (Efron and Tibshirani, 1993) would account for this asymmetry in the sampling distribution, although these confidence intervals would be subject to bias (discussed in Methods section Assessing Uncertainty).

**Figure 8:**
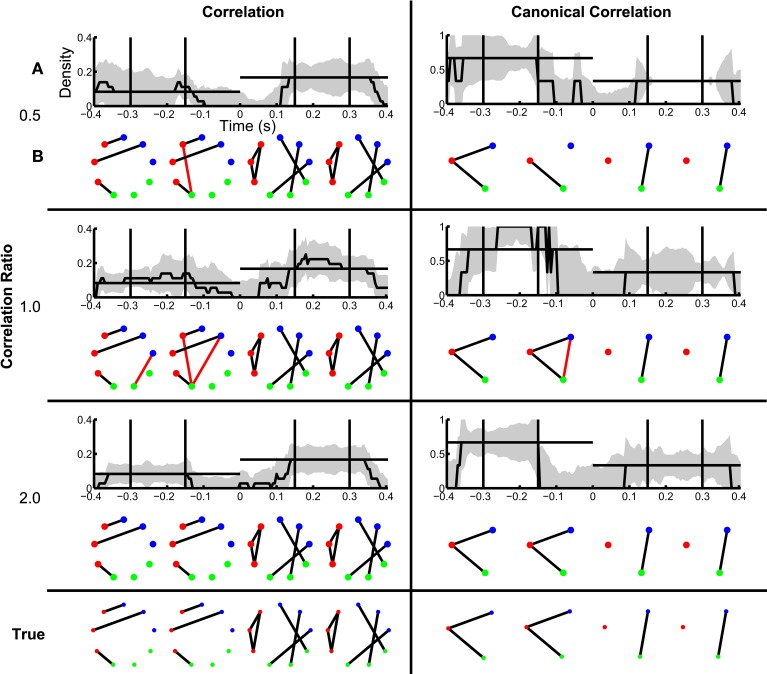
**Dynamic network structure can be detected in the presence of baseline correlations**. Correlation and canonical correlation networks are shown as a function of time with respect to task onset, for simulations with varying correlation ratio. **(A,B)** as in Figure 7, shown for correlation (left) and canonical correlation (right) networks, for three different levels of correlation ratio: 0.5 (top), 1.0 (middle), and 2.0 (bottom).

When constant correlations are introduced, we find results similar to the SNR simulation (Figure 8). Although spurious edges do appear in the correlation networks (see Figure 8B), the inference procedure accurately identifies the true networks both before and after task onset (examples in Figure 8B). In addition, the confidence intervals for the density exclude zero, and include the known density value before and after task onset in some cases (Figure 8A); for the reasons described above, the correlation networks before task onset and the canonical correlations after task onset often do not achieve significant nonzero density. In this way, the establishment of confidence intervals for the aggregate network measure (density) permits identification of dynamic changes in network structure. Surprisingly, in the presence of strong baseline correlations (ratio 0.5, when baseline correlations are twice as strong as trial correlations), the variability of the canonical correlation networks is very small after task onset: all of the bootstrapped networks had one edge, leading to an estimate of zero for the standard error. In this case, the presence of background correlations causes the baseline canonical correlations to be higher, making it more difficult for spurious correlations to appear as false positives in the network estimation. Hence, the presence of background correlations may in some cases make network inferences more robust to noise under this framework.

### ECoG data

**Figure 9:**
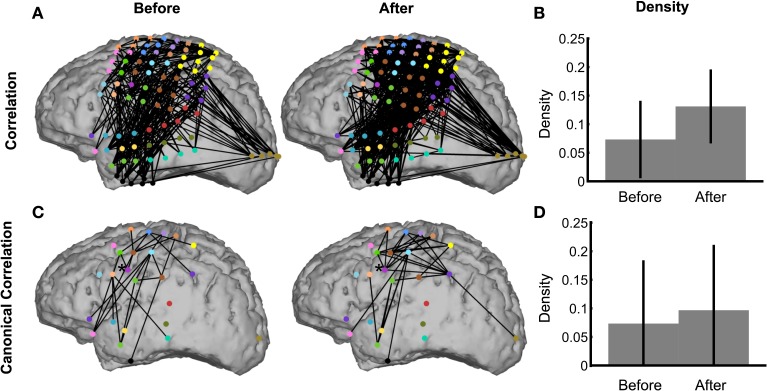
**The network inference procedure facilitates interpretations of real ECoG data**. Binary correlation **(A)** and canonical correlation **(C)** networks were inferred from speech data in the intervals 500 ms before (left) and 500 ms after (right) speech onset. The corresponding densities are significantly nonzero for both correlation networks **(B),** but not for the canonical correlation networks **(D)**. The node colors in **(A)** represent for each electrode the corresponding ROI, shown in **(C)**. Node locations in **(C)** are the average locations of the electrodes corresponding to the ROI. In **(C)**, the node indicated by an asterisk (*) is the ventral primary motor cortex ROI, which is discussed in the text (Methods section ECoG Data).

To demonstrate the use of the network inference procedure on real data, we analyze an ECoG dataset in which a subject read aloud from a teleprompter. Trials are defined by the 500 ms preceding and following speech onset (see Methods section ECoG Data), which includes preparatory activity before speech onset in addition to activity related to motor execution and feedback after speech onset. Visual inspection of the binary correlation networks before and after speech onset is hindered by the large number of edges (Figure 9A), although the network after speech onset appears more dense. This is confirmed by the density plot (Figure 9B), in which the density after speech onset is higher than before, and significantly nonzero. In fact, the density before speech onset is also significantly nonzero, by a narrow margin. The trend of higher density after speech onset also appears in the canonical correlation networks (Figure 9C), although neither achieves significant nonzero density (Figure 9D). With fewer nodes and edges, the canonical correlation binary networks (Figure 9C) are easier to interpret anatomically: for example, the region corresponding to ventral primary motor cortex [indicated with an asterisk (*)] has two edges before speech onset and six edges after speech onset, showing local coupling with premotor and primary motor cortex during motor planning that expands during speech execution to somatosensory cortex and the supramarginal gyrus. These patterns are consistent with speech processing areas identified by fMRI (Hickok and Poeppel, 2007; Price, 2012).

### Advantages of the canonical correlation measure

The results in the previous section illustrate the utility of the network inference procedure for both the correlation and canonical correlation measures. We found that the canonical correlation provides additional robustness to the appearance of spurious edges between individual node pairs. We now consider two additional examples that illustrate the utility of the canonical correlation measure in functional network inference.

#### Canonical correlation improves detectability of weak inter-regional connections

**Figure 10:**
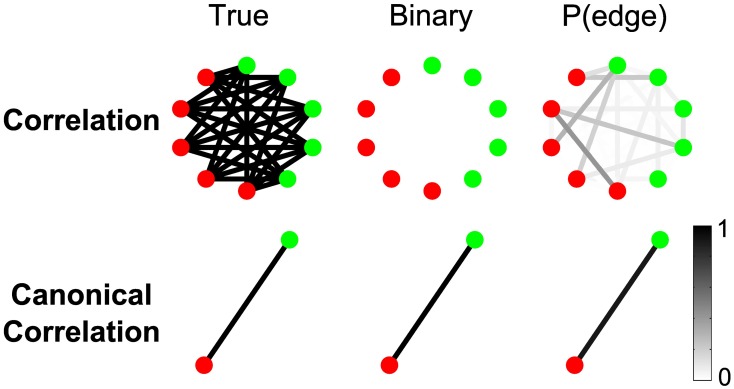
**Canonical correlation improves detectability of weak inter-regional connections**. While the total SNR on each electrode is about 0.14, the correlated signals contributing to each true edge (5 per node) are weak, with SNR of about 0.03. For more details about the simulation, see Results section Canonical Correlation Improves Detectability of Weak Inter-Regional Connections (“Example 1” in Table A1). Shown are the true network (left), binary network (middle), and P(edge) (right) for correlation and canonical correlation networks. In all networks, nodes are color-coded according to region, and edges occur between regions.

One advantage of region-level analyses is that weak connectivity undetectable at the sensor level can be aggregated in such a way as to become detectable at the region level. To illustrate this idea, we constructed a simple scenario with 10 sensors comprising two regions of five sensors each (Figure 10). During trials, every sensor from Region 1 (red nodes in Figure 10) is weakly connected to every sensor from Region 2 (green nodes in Figure 10): while the total SNR on each sensor is approximately 0.14, the correlated signals contributing to each true edge are weak, with SNR of about 0.03. Figure 10 shows that, while the correlations are too weak to be detected in sensor-space networks, in the region-space networks the canonical correlation between the two regions is strong enough to reveal the true edge. There are two reasons for the improved performance of the region-space analysis. First, the weak connections between the sensors are combined in the canonical correlation calculation, increasing the effective SNR and statistical power. Second, the region-space network has many fewer edges (in this case only one), making the FDR correction for multiple comparisons much less severe and allowing higher *p*-values for the test statistics to be classified as edges.

#### Canonical correlation outperforms signal averaging within ROIs

In the fMRI literature, it is common to combine the signals from many image voxels into functional ROIs, each containing a large number of neurons believed to be performing a common function. In studies of speech production, for example, commonly used ROIs include the ventral premotor cortex, which contains neurons that represent syllabic motor programs, the supplementary motor area, which contains neurons that initiate the readout of speech motor programs, and the ventral primary motor cortex, which contains neurons involved in generating commands to the articulatory musculature (Tourville et al., 2008; Peeva et al., 2010; Golfinopoulos et al., 2011). ROI-based analyses can increase statistical power for activity contrasts and connectivity analyses by (1) reducing the number of statistical tests and corresponding correction for multiple comparisons, and (2) averaging many noisy measurements together to form a single, more stable measure for each ROI (Nieto-Castanon et al., 2003).

**Figure 11:**
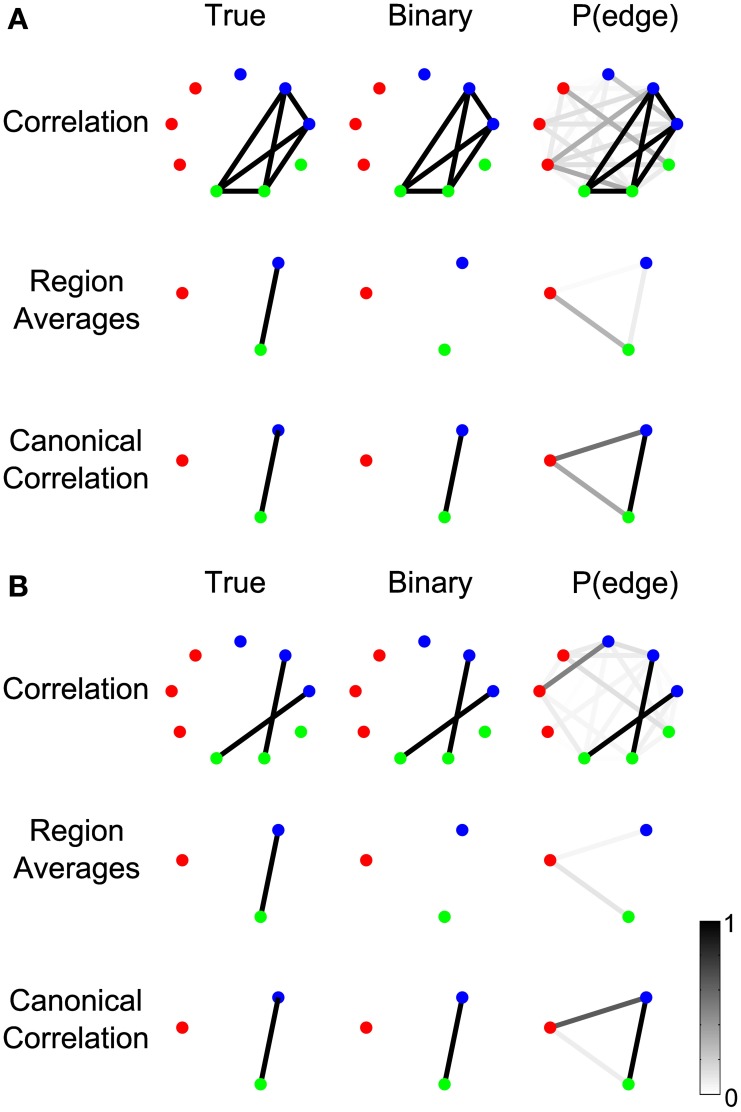
**Canonical correlation outperforms signal averaging within ROIs**. In **(A)**, labeled “Example 2a” in Table A1, the signals on the electrodes within each region precisely cancel. In **(B)**, labeled “Example 2b” in the Table A1, the signals on each edge are independent (so the signals on the electrodes do not precisely cancel in the region averages), but one edge represents a positive correlation and the other edge represents a negative correlation. For more details about the simulations, see Results section Canonical Correlation Outperforms Signal Averaging Within ROIs. **(A,B)** show the true network (left), binary network (middle), and P(edge) (right) for correlation networks (top), networks resulting from averaging the signals on the electrodes within the regions and calculating the correlations between the resulting region-level signals (middle), and canonical correlation networks (bottom).

In the case of fine time-resolution voltage recordings (e.g., scalp EEG or invasive ECoG), the fast dynamics of the signals within a region may not be phase-aligned; therefore, the averaged signals may interfere and cancel in the sum, or the SNR may be reduced. Canonical correlation does not require averaging, and can effectively detect and aggregate signals between sensors even when the sensor signals are not phase aligned. To demonstrate this, we constructed two example scenarios, each containing nine nodes that are split into three regions, of which only two regions are connected by an edge (Figure 11).

In the first scenario (Figure 11A), one signal is shared by two sensors in the green region and two sensors in the blue region, but the sign of the signal is reversed on one sensor in each region such that the average signal within each region is zero. This creates a completely connected subnetwork, where the sensors within each region are perfectly negatively correlated. In this case, averaging the signals within each region cancels the correlated component, resulting in only the noise component. Hence, the correlation between the averaged region signals is zero and no edge is detected (middle row). The canonical correlation network, however, does detect the edge (bottom row), because a change of sign on the individual sensors can be compensated by the coefficients in the linear combination (vectors $\vec{a}$ and $\vec{b}$ in Methods section Computing the Test Statistic).

The second scenario (Figure 11B) illustrates a related point: that averaging signals within regions assumes that the sign of the correlation will be the same for all connections between the regions. In this scenario, one sensor from the green region has a positive correlation with one sensor from the blue region, while a second sensor in the green region has a negative correlation with a sensor from the blue region. At the sensor level, a negative correlation during trials represents an increase in the magnitude of the correlation, so the sensor-level networks detect both edges despite the fact that one reflects a positive correlation and the other reflects a negative correlation (top row). For region level networks, however, canonical correlation detects the true structure (bottom row) while averaging the signals within the region does not (middle row). This is because averaging the signals in the two regions combines the effects of the positively and negatively correlated edges, resulting in a combined signal that is neither positively nor negatively correlated. Hence the edge between the regions is not detected in the region-averaged network (middle row). Canonical correlation, however, is not sensitive to the sign of the correlations between sensors because of the freedom in the linear combination (Methods section Computing the Test Statistic), so it detects the edge in the region-level network (bottom row).

## Discussion

Here we described a method to infer functional networks from time series data recorded from multiple sensors in a task-related paradigm. In doing so, we tracked the changes in dynamic network topology over time, and established measures of uncertainty for both individual edge and aggregate network measures. We also introduced an additional measure of coupling—canonical correlation—which provides a principled approach to aggregate sensor activity, and improves robust detection of network structure. We illustrated the performance of the network inference procedure applied to synthetic data in a variety of simulation scenarios, and verified its utility when used with experimental ECoG data. In this section, we summarize the primary features of this research, mention associated limitations, and suggest avenues for future research.

### Principled choices in functional network analysis

In this manuscript, we described a specific procedure for functional network inference. This procedure can be broken down into several modules that can be individually substituted with other choices without significant modification of the overall procedure. The modules include: (1) choice of trial epochs: here we used two kinds of epochs, 500 ms before and after task onset or 200 ms sliding windows over the duration of the trials; (2) selection of network nodes, either treating each sensor as a separate node or choosing groups of sensors according to, e.g., anatomical region; (3) choice of coupling measure: here we used correlation and canonical correlation; (4) choice of method for estimating the null distribution: here we used bootstrapping on baseline intervals; (5) choice of method for correcting for multiple comparisons, here FDR; and (6) choice of method for estimating uncertainty in edges and network measures: here we used bootstrapping over trials and standard error confidence intervals (see Methods).

The methodology developed here could be extended to accommodate alternative choices. For example, the coupling measure used to define edges could be replaced by a frequency domain measure (e.g., coherence or canonical coherence) or a nonlinear coupling measure (e.g., synchronization likelihood: Stam and Van Dijk, 2002; Pereda et al., 2005). In this case, the same procedures for estimating the null distribution, correcting for multiple comparisons, and estimating edge and network uncertainty can still be used. In summary, the framework discussed here illustrates a specific implementation of a functional network inference procedure that researchers may adapt to suit their own needs.

### Tracking of dynamic network topology over time

As brain activity changes dynamically to achieve specific functions (e.g., to respond to external stimulation), we expect that coupling between the activity from separate brain areas will also change. Because of this, the resulting functional networks—deduced for sensors observing these brain areas—will also change dynamically. To accurately track these dynamic changes requires that we choose analysis intervals of sufficiently short temporal duration. However, choosing a short temporal interval reduces the number of data points employed in the coupling analysis, and therefore reduces the statistical power of any coupling measure. The effect is mitigated for the task-related data considered here. In this case, the multiple repetitions of the task provide increased statistical power. In addition, the multiple trial structure permits a principled resampling procedure. Extending this approach to spontaneous data that lacks a task-related structure would require additional careful considerations, including whether resampling is appropriate.

### Spatial scale

One approach to dealing with issues of spatial scale is to group nodes into interconnected sub-groups after a full network has been constructed (Salvador et al., 2005; Ferrarini et al., 2009; Kolaczyk, 2009; Meunier et al., 2009; Rubinov and Sporns, 2010). These types of approaches can identify functional clusters in networks and were not considered here. However, clustering on sensor-space networks could be used to define ROIs for subsequent ROI-based analyses, suggesting a multi-step procedure that utilizes fine spatial resolution networks to inform networks with coarser spatial resolution.

Note that in situations in which functional regions are known beforehand, using knowledge of ROIs can improve network inference. For example, in Results section Canonical Correlation Improves Detectability of Weak Inter-Regional Connections, we illustrated a situation in which region-level connectivity was too weak to be visible in sensor-space networks, and the greater power of region-level analysis was necessary to detect the connection.

A priori knowledge of ROIs can also be used to perform across-subject analyses in situations when it would otherwise be difficult. For example, in ECoG recordings, the electrode locations typically differ for each subject, but the spatial coverage of the electrodes often overlaps across subjects. Using the same ROIs to construct region-level networks for each subject could facilitate across-subject comparisons. In addition, the data for each ROI could be aggregated across subjects before network construction and aggregate across-subject networks could be estimated. These across-subject networks could be calculated as a function of time during a task, with the goal of detecting dynamic changes in connectivity that are not subject-specific.

The specific coupling measure used here for region-level network inference, canonical correlation, is a particularly robust tool for detecting connectivity between predefined regions. As described above, fMRI studies often average the signals of voxels within functional ROIs. We showed two examples of how averaging signals within an ROI can mask inter-regional correlations (Results section Canonical Correlation Outperforms Signal Averaging Within ROIs) that are detected using canonical correlation. In the first scenario, signals within an ROI are responsive to task onset but with opposite sign; in this case averaging results in a net signal for the ROI that is not responsive to task onset, which in turn results in weak or nonexistent correlations between the ROI and other ROIs involved in the task. This could occur in physiological data in which electrode location relative to an electrical source induces a change in sign of the recorded activity between neighboring electrodes (e.g., Wood et al., 1988). In the second example, two ROIs are both positively and negatively correlated with each other, through different pairs of electrodes. This situation could also occur in real data in which the location of neighboring electrodes may lead to a reversal in sign (Wood et al., 1988; Buzsaki et al., 2012). In this case the averaged signal for each ROI is still responsive to task onset, but averaging the signals masks the positive and negative correlations with other regions. Our simulation results demonstrate that canonical correlation successfully identifies inter-regional correlations in these situations.

One drawback of canonical correlation is that it can depend on the number of signals (electrodes) in the groups (ROIs) being compared: because more signals provide more degrees of freedom for the linear combinations involved in the calculation, canonical correlation values tend to be higher when calculated between ROIs with more component electrodes. Here, the raw canonical correlation value between two ROIs is compared to a baseline distribution calculated on the same two ROIs, so ROIs with more electrodes do not necessarily have a greater probability of edge detection. On the contrary, due to ceiling effects (canonical correlation values are bounded above by one) it may be difficult for ROIs with large numbers of electrodes to achieve significance in comparison of trials to baseline. In our simulations, we considered the case where all ROIs have the same number of electrodes. For the ECoG data, the size of each ROI depended on the number of electrodes that happened to lie over the anatomical ROIs, so ROIs had varying numbers of component electrodes. While ROIs with large numbers of electrodes do have edges in the networks inferred here, it is possible that there are imbalances in the inference that are affecting the resulting networks. Further investigation into differing electrode counts for different ROIs is left for future study.

### Assessment of uncertainty in network edges and aggregate network measures

An important component of the network inference procedure outlined above is the ability to assess uncertainty in network structure and aggregate network measures. To do so, we resampled the task-related data, and established both the probability of appearance for each individual edge, and confidence intervals for the network density. Although we focused on only a single aggregate measure here (the density) this procedure is easily extended to determine confidence intervals for other aggregate network measures (e.g., clustering coefficient, path length, etc.). We note that the resampling procedure described here requires repeated occurrences of the time period of interest, such as occurs in a task-related structure, and may not be appropriate for data lacking this structure. In addition, the resampling procedure is computationally expensive, and for a large number of resamples would require sophisticated computational approaches.

While we focus on brain voltage data (ECoG and EEG) here, there is some precedence of the use of bootstrapping to estimate network variability in the fMRI resting state functional connectivity literature. For example, bootstrapping has been used to test whether seed-based resting state connectivity is dependent on signal stationarity (Chang and Glover, 2010) and phase coupling (Handwerker et al., 2012). Bootstrapping has also been used to test for group differences in functional networks between schizophrenia patients and controls (Sakoglu et al., 2010) and between genders (Kilpatrick et al., 2006). Allen et al. (2014) used bootstrapping to assess the reliability of estimated network states, defined by clustering dynamic networks occurring over the course of an experimental session.

Bootstrapping has also been used in EEG functional connectivity, albeit in the context of network inference. Murias et al. (2007) used bootstrapping to compute *p*-values for pairs of electrodes under the null hypothesis that coherences did not differ between children with attention deficit hyperactivity disorder (ADHD) and controls. These *p*-values were then used to define edges in a functional network, similar to our network inference approach (Methods section Assessing the Significance of the Test Statistic).

### Robustness to persistent correlations

By allowing the null distribution to be different for each potential edge, systematic spatial regularities in the correlations are mitigated. In the case of a speech task, correlations unrelated to speech may exist in the data for many reasons, including the influence of resting state networks, volume conduction, and referencing effects. As long as these regularities are present in the baseline data to the same extent as the task data, these correlations will not appear as edges in the networks since the correlations during speech must be stronger than the correlations during the baseline silent periods in order to achieve significance. Volume conduction due to deep subcortical sources of electrical activity may also confound inference of functional networks. For the task-related data of interest here, a deep subcortical source not present during silence that emerges during the task could introduce correlations during speech that would be interpreted by the procedure as edges. These edges are spurious, in that the edge does not represent correlated cortical activity appearing locally at each sensor. While this is a valid concern, strategies exist to mitigate volume conduction effects, including re-referencing procedures in scalp EEG. Moreover, invasive recording modalities—such as the ECoG—are proposed to be relatively insensitive to distant electrical sources (Zaveri et al., 2009).

### Scalability and challenges related to experimental data

One of the key difficulties in network construction is the issue of multiple comparisons: a test statistic is computed for each pair of electrodes, so a 100-electrode network (typical in ECoG recordings) represents 4950 statistical tests. Here, we account for multiple comparisons using the FDR procedure, which controls the proportion of false positive edges relative to the total number of detected edges. This highlights connections between nodes that are extreme relative to the baseline period: the *p*-values for the test statistics need to be very small in order to be counted as edges. This approach puts more interpretative power on the existence of edges than their absence: if an edge exists in an inferred network it reflects a strong difference between trial and baseline coupling, but if an edge does not exist there might be a difference that was not strong enough to be classified as significant. This could cause problems in situations where the power of the statistical test is different for different pairs of electrodes, for example if the SNR varies considerably across the set of electrodes. In this case, the variance of the test statistics may be higher in the baseline condition for pairs of electrodes with low SNR, making it more difficult for the trial period coupling to achieve low *p*-values compared to pairs of electrodes with high SNR. This could ultimately lead to increased false negative edges in the network for edges involving electrodes with relatively low SNR. Hence it is important to take caution in the interpretation of networks in the context of varying SNR, especially with respect to absent edges, since the FDR procedure does not control for false negatives. Note also that the FDR procedure assumes independence between the component tests (Benjamini and Hochberg, 1995). It is possible that a network structure introduces dependencies between the test statistics calculated for each potential edge. If this is the case, a corrected FDR procedure or another control for multiple comparisons may be more appropriate (Dudoit and Laan, 2008).

Additionally, we note that the uncertainty measures we describe are to be interpreted in terms of comparisons between a single epoch and a baseline period, not in terms of comparisons between epochs. Care should be taken in claiming significance in changing network structure through multiple time epochs. The fact that one epoch shows a significant difference in network structure from baseline while another epoch shows none, does not necessarily indicate a significant difference in network structure between epochs. In order to quantify the significance of changes across epochs, similar methods can be developed to those discussed here.

As high density multi-sensor data becomes increasingly common, techniques to characterize these data—and notions of uncertainty in these characterizations—become essential. In this paper we described a general framework for the inference of functional networks from task-related, multi-sensor data. We proposed a principled approach for assessing uncertainty in network edges and aggregate network measures, as well as a technique to aggregate sensor activity and improve detection of network structure. Within this framework, we made specific choices to construct the functional networks. However, the framework is general and adaptable to choices optimized to specific recording modalities and research questions.

### Conflict of interest statement

The authors declare that the research was conducted in the absence of any commercial or financial relationships that could be construed as a potential conflict of interest.

## Appendix

### Construction of simulated data

The simulation consisted of 600 s of synthetic recordings from nine sensors at a sampling frequency of 1200 Hz. The nine sensors were split into three regions, consisting of three sensors each. The first 400 s of the data were a “baseline period” containing no trials, followed by 100 trials lasting 1 s each, separated by 1 s. The total signal was a sum of four subcomponents: pink noise, white noise, constant correlations, and trial correlations (Figure 1D). The sensor-space and region-space connectivity networks existing during the trials were the same for all simulations, consisting of two different network topologies present for the first and second halves of the trials, called the Before and After periods, respectively, (Figure 1A). The magnitudes of the different components and the strength of the correlations were manipulated in a series of scenarios designed to test the effects of the SNR and constant background correlations on the ability of our network analysis methodology (described in Methods section Construction of Functional Networks) to detect task-related changes in the correlation structure.

While we intend to make the code available online, it will not be posted before the publication.

#### Pink noise

Pink noise, *P*(*t*), was generated for each sensor by convolving white noise with a Gaussian kernel of standard deviation 5 ms. The spectrum of the resulting signal (Figure 1C, top) roughly approximated the decrease in power with frequency commonly observed in neuronal voltage recordings (Miller et al., 2009; He et al., 2010). After filtering, the signal was normalized by the standard deviation, resulting in a signal with unit variance.

#### White noise

The white noise component, *W*(*t*), for each sensor had variance 0.1.

#### Constant correlations

Constant correlated structure was included by adding a single 2–50 Hz signal lasting the entire duration of the recording, *C*(*t*), to every sensor. Because the same signal was added to all sensors, the network topology corresponding to this signal is a complete graph, in which every node is connected to every other node. In order to vary the strength of the constant correlations while holding total variance and spectral characteristics constant, an additional 2–50 Hz signal, *B*(*t*), uncorrelated between sensors, was added to each sensor. Signals in the desired passband were generated by filtering white noise using a zero-phase 4th order Butterworth filter (Matlab filtfilt function). After filtering, the signals were normalized to have unit variance and multiplied by a gain factor that varied by simulation (see Appendix section Simulation Scenarios).

#### Trial correlations

Task-related correlation structure was introduced into each sensor of the synthetic data in the 8–25 Hz frequency range in order to introduce pre-defined network structures in the first 500 ms (“Before” task onset) and the second 500 ms (“After” task onset) of the trials (Figure 1A). In the true Before network, edges exist between sensors 1 and 9, 2 and 8, and 3 and 4. This corresponds to a region-space network in which Region 1 (red) is connected to both Region 2 (green) and Region 3 (blue). In the true After network, the sensors in Region 1 (red nodes) are completely connected (cyan edges), and Regions 2 (green nodes) and 3 (blue nodes) have three pairs of connected sensors between them (magenta, yellow, and green edges). The region topology therefore contained only one edge, between Regions 2 and 3.

Figure 1B shows how the trial correlations between sensors were introduced into the signal, using the After network to illustrate. The 8–25 Hz component was constructed to have constant instantaneous variance during the entire dataset, switching from uncorrelated 8–25 Hz noise outside of trials and transitioning to correlated 8–25 Hz noise during trials. To achieve this, three signals were summed at each sensor: one uncorrelated signal lasting the entire duration of the data that tapers away during the correlated portions of the trials, (Figure 1B, middle), one correlated signal corresponding to the Before network that appears only during the first half of the trials (not shown), and one correlated signal corresponding to the After network that appears only during the second half of the trials (Figure 1B, left). The signal added to each sensor was chosen to introduce a fixed network topology (the colored edges in Figure 1A, corresponding to colored traces in Figure 1B) of the correlations during the trials that would not exist in the baseline period.

The window functions governing the transition between uncorrelated activity and correlated activity (*W*_*U*_(*t*) and *W*_*T*_(*t*), respectively; Figure 1B, top) were chosen so that the instantaneous variance of the signal (Figure 1B, bottom) would stay constant during trials. In particular, the correlated signal was windowed by the square root of a Gaussian kernel with σ = 50 ms and μ = 250 ms:

$$W_{T}(t)=\sqrt{\frac{1}{\sigma \sqrt{2\pi }}e^{\frac{-{\left(t-\mu \right)}^{2}}{2\sigma^{2}}}}$$

The uncorrelated signal was windowed by:

$$W_{U}(t)=\sqrt{1-\frac{1}{\sigma \sqrt{2\pi }}e^{\frac{-{\left(t-\mu \right)}^{2}}{2\sigma^{2}}}}$$

These choices resulted in correlated and uncorrelated signals, *T*(*t*) and **U**(*t*), respectively, having instantaneous variances that evolve in time according to a Gaussian profile and one minus that Gaussian profile:

$$\begin{array}{l} \;Var(T(t))=\frac{1}{\sigma \sqrt{2\pi }}e^{\frac{-{\left(t-\mu \right)}^{2}}{2\sigma^{2}}}\\ Var(U(t))=1-\frac{1}{\sigma \sqrt{2\pi }}e^{\frac{-{\left(t-\mu \right)}^{2}}{2\sigma^{2}}}. \end{array}$$

Hence the combined signals had unit variance (Figure 1B, bottom). The combined signal was then multiplied by a gain factor that varied by simulation (see section Simulation Scenarios). In the resulting signal, the total variance of the correlated signal, *T*(*t*), over the entire trial (for electrodes with at least one edge in both the before and after networks) was about one quarter of the total variance, the other three quarters of the total variance being contributed by *U*(*t*).

#### Simulation scenarios

A series of simulation scenarios were constructed in order to investigate the performance of the network analysis techniques under different conditions related to the SNR and to the ratio of trial correlations to constant correlations.

The first set of simulations tested the effects of SNR on inferred trial networks in the absence of constant correlations (Table A1, SNR simulations). SNR was defined as:

$$SNR=\frac{Var(T)}{Var(U)\;+\;Var(C)\;+\;Var(B)\;+\;Var(W)\;+\;Var(P)}$$

where the variances are calculated over time during the trials (both the Before epoch and the After epoch), in which the trial correlation component *T*(*t*) has nonzero variance. Note that in all simulations, the white noise component *W*(*t*) had a variance of 0.1 and the pink noise component *P*(*t*) had a variance of 1. In these simulations, *C*(*t*) and *B*(*t*) were set to zero and the gain on the 8–25 Hz component (*T*(*t*) + *U*(*t*)) was varied to achieve different SNR values (Table A1).

**Table A1:** **Parameters for simulation scenarios**.

**Simulation**	**8–25 Hz variance**	**2–50 Hz variance**		**SNR**	**Correlation ratio**	**Results figures**
		**Uncorrelated**	**Correlated**			
SNR 1	0.00	0	0	0.00	.	Figures 3, 4, 7
SNR 2	0.26	0	0	0.05	.	Figures 3, 4, 7
SNR 3	0.63	0	0	0.10	.	Figures 3, 4, 7
SNR 4	1.20	0	0	0.15	.	Figures 3, 4, 7
Ratio 1	1.00	0	0.5	0.11	0.5	Figures 5, 6, 8
Ratio 2	1.00	0.25	0.25	0.11	1.0	Figures 5, 6, 8
Ratio 3	1.00	0.37	0.13	0.11	2.0	Figures 5, 6, 8
Example 1	1.00	0	0	0.14	.	Figure 10
Example 2A	1.00	0	0	0.14	.	Figure 11A
Example 2B	1.00	0	0	0.14	.	Figure 11B

*Here we list the settings utilized in the simulation scenarios considered in Results. Figures illustrating the inferred functional networks for each scenario are listed in the last column*.

The second set of simulations tested the effects of constant background correlations on the inferred trial networks (Table A1, “Ratio” simulations). By using the same gains for the 8–25 Hz component, *T*(*t*) + *U*(*t*), and the 2–50 Hz component, *C*(*t*) + *B*(*t*), for all of these simulations, the SNR was held the same. What varied in these simulations was the relative variances of *C*(*t*) and *B*(*t*). This resulted in simulations in which the ratio of the variance of the trial correlations *T*(*t*) and constant correlations *C*(*t*) varied:

$$CorrelationRatio=\frac{Var(T)}{Var(C)}$$

where, as above, the variances are calculated over time during the trials. Note that *Var*(*T*) is about one quarter of the total variance of the 8–25 Hz component.

Finally, simulations were constructed for two example scenarios to illustrate different features of the analysis. These examples are described in more detail in the Results (section Canonical Correlation Outperforms Signal Averaging Within ROIs).

### Sparsity of networks and null distribution resolution effects

The smallest possible *p*-value in the described bootstrap procedure, *p*_(1)_, is bounded below by the number of bootstrap samples used to calculate the null distribution, here 1/1000 for the simulated data. In a typical network, there may be many potential edges whose test statistics fall above the highest sample in their respective empirical null distributions, leading to a set of *p*-values *p*_(1)_, …, *p*_(*k*_min_)_ equal to 1/1000. If the total number of these, *k*_min_, satisfies

$$p_{\left(k_{\min}\right)}=\frac{1}{1000}\leq \frac{qk_{\min}}{N_{MC}}$$

then all of the tests 1,…, *k*_min_ will be considered significant. If *p*_(*k*_min_)_ > *qk*_min_/*N*_*MC*_, all of the tests will be considered insignificant and the network will be reported as empty. This characteristic, resulting from the interaction of the FDR procedure with empirical estimation of the null distribution, imposes a lower bound on the sparsity of networks that can be detected: anything below this sparsity level will be reported as an empty network. Analytically, the smallest number of edges that can be detected is:

$$\frac{N_{MC}}{qN_{bs}}$$

where *N*_*bs*_ is the number of bootstrap samples used for the estimation of the null distributions. Note that this formula also depends on *N*_*MC*_, the number of potential edges in the network, which scales with the square of the number of electrodes. Hence this issue becomes more serious as the size of the networks increases.

There are a variety of possible ways to deal with this issue. The first is to reduce the number of nodes in the networks (hence reducing the number of possible edges) by defining the networks on a larger spatial scale, for example by using the ROI-based analysis using canonical correlation described here. With fewer possible edges, it can become computationally tractable to use enough bootstrap samples to detect even a single edge. When reducing the number of nodes is not possible, a second option is to increase the number of bootstrap samples so that the minimum number of detectable edges is acceptably small. This is a reasonable first approach, since in many applications the networks are sufficiently dense to exceed the threshold. If the resulting inferred networks are not empty, no further analysis is needed. If the networks are empty, however, it could either be because there really is no coupling or because the number of true edges is smaller than the detectable amount. In this case, two other options are available: (1) estimate the null distributions parametrically, or (2) estimate the tails of the null distributions above the highest bootstrap sample using extreme value theory. In the case of correlations, the Fisher transform of the correlation is well approximated by a Gaussian distribution (Fisher, 1915), so the parametric approach is possible. When the sampling distribution of the test statistic is not well behaved, an approach to estimating the *p*-values only in the tails of the distribution based on extreme value theory may be used (Hill, 1975; Pickands, 1975; Smith, 1987).

For the experimental data used here, we increased the number of bootstrap samples in order to detect networks with very few edges. Specifically, the correlation networks had 90 nodes and we used 20025 bootstrap samples, so the smallest number of edges that could be detected was

$$\frac{90*89/2}{0.05*20025}=4$$

For the canonical correlation networks, we had 25 regions and used 6000 bootstrap samples, so that we could detect a network with a single edge. Note that by reducing the number of nodes, canonical correlation networks require many fewer bootstrap samples.

### Network inference algorithm structure including uncertainty estimation

In this section we provide a description in pseudo-code of the uncertainty estimation algorithm. Symbols are defined at the end of this section.

**Network estimation with uncertainty:**

- **Estimate the null distribution from baseline data (the same null distribution is used for the observed network and the bootstrapped networks):**

-  For *j* = 1 : *N*_*bs*_,

-    *I*_*baseline*_ ← a set of *L* indices chosen uniformly at random (with replacement) from 1 : *K*

-    *Sample*_*bs*_ ← the data for baseline intervals corresponding to *I*_*baseline*_ : (*T* × *N* × *L*)

-    $\hat{x}$_*bs*_ (*j*,:,:) ← (1 × *N* × *N*) or (1 × *R* × *R*): the test statistic at each edge calculated from *Sample*_*bs*_

-  End

- **Resample trials to compute bootstrap networks and network measures**

-  For *i* = 1 : *N*_*us*_,

-    **Select a sample of trials over which to compute a surrogate network:**

-      *I*_*us*_ ← a set of *L* indices chosen uniformly at random (with replacement) from 1 : *L*

-      *SampleI* ← (*E* × *T* × *N* × *L*): the data for trials corresponding to *I*_*us*_

-    **Calculate the true $\hat{x}$ for the sample:**

-      $\hat{x}$_*i*_ ← (*E* × *N* × *N*) or (*E* × *R* × *R*): the test statistic at each edge calculated from *SampleI* for each epoch

-    **Compare** $\hat{x}$_*i*_, (*E* × *N* × *N*) or (*E* × *R* × *R*), **at each edge to the empirical null distribution given by** $\hat{x}$_*bs*_, (*N*_*bs*_ × *N* × *N*) or (*N*_*bs*_ × *R* × *R*):

-      For epoch *e* = 1: *E*,

-        For node *n*_1_ = 1 : *N* (or 1 : *R*)

-          For node *n*_2_ = *n*_1_ + 1 : *N* (or *n*_1_ + 1 : *R*)

-            $p\left(e,\;n_{1},\;n_{2}\right)\leftarrow \frac{\#\left({\hat{x}}_{bs}\left(:,\;n_{1},\;n_{2}\right)>{\hat{x}}_{i}\left(e,\;n_{1},\;n_{2}\right)\right)}{N_{bs}}$, where # denotes a count of the number of elements satisfying the expression in parentheses

-            If *p*(*e, n*_1_, *n*_2_) == 0 then *p*(*e, n*_1_, *n*_2_)1/*N*_*bs*_

-          End

-        End

-      End

-    **Use FDR procedure to identify significant edges:**

-      For epoch *e* = 1 : *E*,

-        *p*_(.)_ ← (1 × *N*_*MC*_): Sorted *p* for the epoch *e*, for all edges

-        *k* ← the highest *k* such that *p*_(k)_  ≤ $\frac{qk}{N_{MC}}$

-        *threshold* ← *p*_(*k*)_

-        *Net* (i,e,:,:) ← (1 × 1 × *N* × *N*) or (1 × 1 × *R* × *R*): *p*(*e*,:,:) ≤ *threshold* (binary network consisting of all edges with a *p*-values less than *threshold*)

-      End

-    **Calculate aggregate network statistics:**

-      For epoch *e* = 1 : *E*,

-        *Density*(i,e) ← $\frac{\#\;edges\;in\;Net\left(i,e,:,:\right)}{N_{MC}}$

-      End

-    End

-    Return *Net* (*N*_*us*_ × *E* × *N* × *N*) and *Density* (*N*_*us*_ × *E*)

- **To calculate the probability of an edge for each epoch and edge:**

- For epoch *e* = 1 : *E*,

-  For node *n*_1_ = 1 : *N* (or 1 : *R*)

-    For node *n*_2_ = *n*_1_ + 1 : *N* (or *n*_1_ + 1 : *R*)

-      *P*_*edge*_ (*e, n*_1_, *n*_2_) ← $\frac{\#\;edges\;in\;Net\left(:,e,n_{1},n_{2}\right)}{N_{us}}$

-    End

-  End

- End

- **To calculate alpha-level confidence intervals on the density for each epoch:**

- For epoch *e* = 1 : *E*,

-  *ObservedDensity*(*e*) ← the density of the observed network (calculated using all trials)

-  *StandardError*(*e*) ← the standard error of *Density*(:, *e*)

-  *LowerBound*(*e*) ← *ObservedDensity*(*e*) − 1.96 * *StandardError*(*e*)

-  *UpperBound*(*e*) ← *ObservedDensity*(*e*) + 1.96**StandardError*(*e*)

- End

**Constants:**

*N*_*us*_ : the number of bootstrap samples for uncertainty estimation

*E*: the number of epochs

*T*: the number of time points in each epoch

*N*: the number of sensors

*R*: the number of regions

*L*: the number of trials

*K*: the number of baseline intervals (K > L)

*N*_*bs*_ : the number of bootstrap samples for null distribution estimation

*q*: The FDR threshold for the number of false positives (here, 0.05)

*N*_*MC*_ : the number of comparisons performed for each network, equal to the number of possible edges, *N*^*^ (*N* − 1)/2 or *R*^*^ (*R* − 1)/2

α: the desired type-I error rate for confidence intervals on aggregate statistic.
